# Supplementary material for: The Development of a Simple Projection-Based, Portable Olfactory Display Device
Source: Sensors (Basel). 2023 May 30;23(11):5189. doi: 10.3390/s23115189 (PMC10255882; doi:10.3390/s23115189)
Supplement: Supplementary file 1 [file sensors-23-05189-s001.zip › Post-experiment Questionnaire 36.pdf]

Post-experiment Questionnaire

1. What is your reference number?

36

2. On a scale of 10, please rate how comfortable you feel when you are using this type of scent display. (1 is very poor and 10 is very good)

8

3. What would you choose between a projection-based display (the one you experienced just now) and a wearing type display (e.g. scent releasing masks and collars)?

□Projection-based display □Wearing type display □Not sure

4. Please give reasons for your choice of your previous answer.

With the display used in the experiment, it would sometimes be hard to catch the sent. I believe a wearing type display would be more effective

5. Will you purchase this type of scent display device in the future? If so how much would you like to pay?

□No, I will not purchase it. □Yes, I would like to pay if the price is under 300 pounds

□Yes, I would like to pay if the price range is between 300 to 500 pounds.

□Yes, I would like to pay if the price range is between 500 to 1000 pounds.

□Yes, I would purchase it even if it is above 1000 pounds.

6. What do you think about the experiment? Do you have anything to say to help us improve our results? Please specify below.

The experiment went well, the scent were a bit hard to guess but I believe it went well
