# Supplementary material for: The Development of a Simple Projection-Based, Portable Olfactory Display Device
Source: Sensors (Basel). 2023 May 30;23(11):5189. doi: 10.3390/s23115189 (PMC10255882; doi:10.3390/s23115189)
Supplement: Supplementary file 1 [file sensors-23-05189-s001.zip › Pre-experiment Questionnaire 50.pdf]

Pre-experiment questionnaire

1. What is your reference number?

50

2. Please specify your gender.

☐Male ☐Female ☐Prefer not to say

3. Please specify your age group.

☐18-24 ☐25-34 ☐35-44 ☐45-54 ☐55-65 ☐Prefer not to say

4. How good do you think you sense of smell is? Please rate it from 1 to 10 where 1 is very poor and 10 is very good.

7
