# Supplementary material for: The Development of a Simple Projection-Based, Portable Olfactory Display Device
Source: Sensors (Basel). 2023 May 30;23(11):5189. doi: 10.3390/s23115189 (PMC10255882; doi:10.3390/s23115189)
Supplement: Supplementary file 1 [file sensors-23-05189-s001.zip › Results Table.pdf]

Experiment

Procedure:

1. Pre experiment questionnaire
2. Turn on the ventilation system for 2-3 minutes.
3. Set up the experiment and running at further distances, time the moment when they smell it and the moment they recognize it, ask what intensity out of 10 would they reckon. If time exceeds 3 minutes and they still have not smelled anything, end this session
4. Turn on ventilation system again
5. Change the intensity and repeat step 2 and 3
6. Change the smell and repeat step 2 to 4.
7. Set the equipment to be closer from the user
8. Repeat step 2 and 3 for last smell.
9. Final questionnaire.
10. Smell coffee (beans) in between each scent.

- Part 2: Aroma intensity range testing (Further setting 80cm)

Reference number	Actual odour type	Ethanol intensity	Recognized odour type by volunteer	Odour intensity reckon by volunteer	Time to smell the odour (s)	Time to recognize the odour (s)
36	Pepper Mint	50%	Flowery, Fruity	3	12.84	/
	Pepper Mint	75%	Alcoholic, Spice, Pepper, Antiseptics	5	31.93	/
	Cinnamon	50%	Alcoholic, not sweet, Fruity, Lemons	8	29.63	/
	Cinnamon	75%	Pinetree, Spicy, not sweet, coughing Medicine,	2	43.50	/
50	Lime	50%	Tea tree, Fresh,	3	57.42	/
	Lime	75%	Tea tree, spicy, citric fruit, Lemon	6	51.89	171.29
	Cinnamon	50%	Sweet, fruity, heavier than the first one	5	43.03	/

	Cinnamon	75%	Sweet, Spice like Vanilla, Cinnamon	8	68.91	138.1
30	Pepper Mint	75%	Pepper Mint	8	51.60	51.60
	Pepper Mint	50%	/	4	37.68	/
	Cinnamon	75%	Sweet, Minty, spicy, Cinnamon	3	64.53	126.02
	Cinnamon	50%	/	6	39.31	/
12	Lime	75%	Sour, Coke, Spicy, Lemon	8	36.31	137.13
	Lime	50%	/	5	25.96	/
	Cinnamon	75%	Coke, Flowery	3	53.18	/
	Cinnamon	50%	Coke, Sweet, Fruity, Sour, Lemon,	7	18.04	/

Aroma intensity range testing (Close setting 55cm)

Reference number	Actual odour type	Ethanol intensity	Recognized odour type by volunteer	Odour intensity reckon by volunteer	Time to smell the odour	Time to recognize the odour
36	Lime	50%	Sweet, Fruity, lemon	7	29.30	144.62
	Lime	75%	/	2	44.71	/
50	Pepper Mint	50%	Sweet, Mint	8	72.39	92.44
	Pepper Mint	75%	/	3	22.84	/
30	Lime	75%	Coke, Sweet, Fruity,	1	133.98	/
	Lime	50%	Coke, Spicy, Sweet, Soft Mint, Lemon	2	51.03	/
12	Pepper Mint	75%	Coke, Sour, Cool, Mint	5	30.45	111.06
	Pepper Mint	50%	/	8	54.69	/

All intensity of reported smell measurements are taken on a scale of 10 with 1 represents very poor and 10 represents very strong.
